# A Prognostic Nomogram Model Based on mRNA Expression of DNA Methylation-Driven Genes for Gastric Cancer

**DOI:** 10.3389/fonc.2020.584733

**Published:** 2020-11-24

**Authors:** Zuhua Chen, Bo Liu, Minxiao Yi, Hong Qiu, Xianglin Yuan

**Affiliations:** Department of Oncology, Tongji Hospital, Tongji Medical College, Huazhong University of Science and Technology, Wuhan, China

**Keywords:** gastric cancer, mRNA expression, DNA methylation, nomogram, prognostic model

## Abstract

**Purpose:**

The exploration and interpretation of DNA methylation-driven genes might contribute to molecular classification, prognostic prediction and therapeutic choice. In this study, we built a prognostic risk model *via* integrating analysis of the transcriptome and methylation profile for patients with gastric cancer (GC).

**Methods:**

The mRNA expression profiles, DNA methylation profiles and corresponding clinicopathological information of 415 GC patients were downloaded from The Cancer Genome Atlas (TCGA). Differential expression and correlation analysis were performed to identify DNA methylation-driven genes. The candidate genes were selected by univariate Cox regression analyses followed by the least absolute shrinkage and selection operator (LASSO) regression. A prognostic risk nomogram model was then built together with clinicopathological parameters.

**Results:**

5 DNA methylation-driven genes (*CXCL3*, *F5*, *GNAI1*, *GAMT* and *GHR*) were identified by integrated analyses and selected to construct the prognostic risk model with clinicopathological parameters. High expression and low DNA hypermethylation of *F5*, *GNAI1*, *GAMT* and *GHR*, as well as low expression and high DNA hypomethylation of *CXCL3* were significantly associated with poor prognosis rates, respectively. The high-risk group showed a significantly shorter prognosis than the low-risk group in the TCGA dataset (HR = 0.212, 95% CI = 0.139–0.322, P = 2e-15). The final nomogram model showed high predictive efficiency and consistency in the training and validation group.

**Conclusion:**

We construct and validate a prognostic nomogram model for GC based on five DNA methylation-driven genes with high performance and stability. This nomogram model might be a powerful tool for prognosis evaluation in the clinic and also provided novel insights into the epigenetics in GC.

## Introduction

Gastric cancer (GC) is the third leading cause of cancer-related death and the fifth most common cancer worldwide (1). In the past decades, despite important progress in comprehension of pathology and molecular features, and in development of therapeutic target such as HER2, many patients were diagnosed with inoperable GC with unfavorable overall survival (2). In the era of precision medicine, omics analysis based on DNA, RNA and protein of GC tissues have revealed molecular classifications associated with diagnosis and prognosis (3–5). However, the application of such biomarkers or classifications on daily practice remains challenging to date (6). Hence, diagnostic and prognostic models based on molecular signature and clinical features of patients with GC have important practical value.

The epigenetic modification of nucleic acids, including DNA methylation, histone acetylation, microRNAs, and noncoding RNA, plays an important role in genome stability and gene regulation (7). Various cancers were characterized with the aberrant DNA methylation, such as hypomethylation of oncogene and hypermethylation of suppressor gene, which was involved in tumorigenesis, heterogeneity and therapeutic resistance (8). Therefore, the exploration and interpretation of DNA methylation-driven genes might contribute to molecular classification, prognostic prediction, and therapeutic choice. The prognostic value of MGMT promoter methylation in patients with high-risk glioma treated with radiotherapy and temozolomide highlighted the application feasibility of DNA methylation in clinical implementation (9). In patients with GC, previous studies have revealed that DNA methylation could serve as molecular biomarkers in helicobacter pylori infection, cancer occurrence, and prognosis (10). Meanwhile, with the rapid development of high-throughput sequencing, genome-wide profiling provides us more individualized and systematic evaluation of DNA methylation in cancer (11, 12). However, the molecular mechanisms underlying gene-expression regulated by DNA methylation is unclear, and the diagnostic and prognostic value of these DNA methylation-driven gene remains to be future explored.

Previous studies have reported several prognostic implications in GC, such as the DNA methylation status of nuclear element-1, and the expression of *CLIP4* methylation-associated genes (13, 14). However, due to the lack of simultaneously transcriptomic and DNA methylation profiles analysis, as well as an easy-to-use and quantitative evaluation criterion, these findings are still far from clinical application. In the present study, we constructed a prognostic model for patients with GC *via* integrating analysis of the transcriptome and methylation profile, combined with clinicopathological characteristics. Our findings will contribute to improve the prognosis assessment of GC.

## Materials and Methods

### Patients and Samples

A total of 450 RNA-sequencing profiles (415 GC samples and 35 adjacent samples), 397 DNA methylation-sequencing profiles and corresponding clinicopathological information of 415 patients with GC were downloaded from The Cancer Genome Atlas (TCGA) (https://portal.gdc.cancer.gov/, up to April 20, 2020). Among 415 GC patients, 397 patients had both mRNA expression (Illumina RNA Sequencing platform) and DNA methylation data (Illumina Infinium Human Methylation 450 platform). Patients with overall survival (OS) less than 30 days were removed and resulted with 373 patients. In total, 346 patients had matched transcriptomic data, DNA methylation data and clinical outcomes (OS ≥ 30 days). To validate the relevance between expression of DNA methylation-driven genes and overall survival of patients, we downloaded gene expression profiles of GSE14210 (n = 145), GSE15459 (n = 200), GSE22377 (n = 43), GSE29272 (n = 268), GSE51105 (n = 94) and GSE62254 (n = 300) microarray dataset and corresponding clinical characteristics from the Gene Expression Omnibus (GEO) database.

### Differential Expression Analysis and Survival Analysis of Patients With GC

To identify differentially expressed genes (DEgenes) in GC, differential expression analysis was performed in 415 tumor samples and 35 normal samples from TCGA using t-test followed by p value adjustment with “Benjamini-Hochberg” method. DEgenes were defined as the adjusted p value (p-adjust) < 0.05 and |log_2_ fold change (FC) |> 1. Next, survival analysis by univariate Cox regression was performed to uncover the survival-associated genes (Survgenes) in GC patients. The best cutoff value for each gene was determined by survminer package and the significant Survigenes were defined as p value < 0.001.

### The Identification of DNA Methylation-Driven Gene

The aberrant expression of these DNA methylation-driven genes may be driven factors in the initiation and progression of tumors (15). Briefly, the mean DNA methylation Beta value for all CpG sites in the promoters of certain gene was calculated as the DNA methylation value for the gene. Gene expression and DNA methylation data were automatically matched to identify transcriptionally predictive DNA methylation events. Correlation analysis between gene expression and DNA methylation level were performed in 397 GC samples (16). Methylation-associated genes (Methygenes) were defined as |Coef| > 0.5 and p value < 0.001.

Candidate genes were selected by Venn diagram, only genes meeting the criteria of significance in the differential expression, survival and methylation correlation analyses were chosen for downstream analysis. In order to narrow down the DNA methylation-driven genes significantly associated with prognosis, we utilized the least absolute shrinkage and selection operator (LASSO) analysis, which is a regression analysis method considering both regularization and variable selection (17). After the identification of 5 DNA methylation-driven genes, we performed external validation of the outcome differences between high expression and low expression patients through Kaplan-Meier survival plots (http://kmplot.com/).

### Establishment of the Risk Score Prediction Model

A risk score prediction model was constructed based on the expression levels of the DNA methylation-driven genes filtered by LASSO, which was weighted by coefficients of multivariate Cox regression. The risk score that output the largest χ² value in the Mantel-Cox test was defined as the optimal cutoff value, which stratified GC patients into high-risk and low-risk groups. The exact risk score was calculated according to the following formula. Risk score = β _gene1_ × expression _gene1_ + β _gene2_ × expression _gene2_ + β _gene3_ × expression _gene3_ + β _gene4_ × expression _gene4_ + β _gene5_ × expression _gene5_ + β _gene1_ × methylation _gene1_ + β _gene2_ × methylation _gene2_ + β _gene3_ × methylation _gene3_ + β _gene4_ × methylation _gene4_ + β _gene5_ × methylation _gene5_. We next evaluated outcome differences between high-risk and low-risk patients through Kaplan-Meier survival plots, and evaluated time-dependent receiver operating characteristic (ROC) curves to examine the predictive efficacy of the DNA methylation-driven gene risk model.

### The Design and Validation of the Nomogram

To test the predictive efficacy of the risk score model alone and in combination with other clinicopathological characteristics (including the age, sex, histologic grade, TNM stage and family history) of patients with GC, we performed survival analysis with the clinicopathological parameters. A prognostic nomogram consist of the five methylation-driven genes and available clinicopathological parameters was generated *via* the rms R package (18). Validation of the nomogram contained calibration and discrimination. The calibration was measured by the distance between the predicted probabilities and the 45-degree line, which represented the best prediction. The discrimination, namely the predictive accuracy of a nomogram, was assessed by a concordance index (c-index), which quantified the concordance level between predicted and actual probabilities.

## Results

### The Clinicopathological Characteristics of Enrolled GC Patients

**Figure 1:**
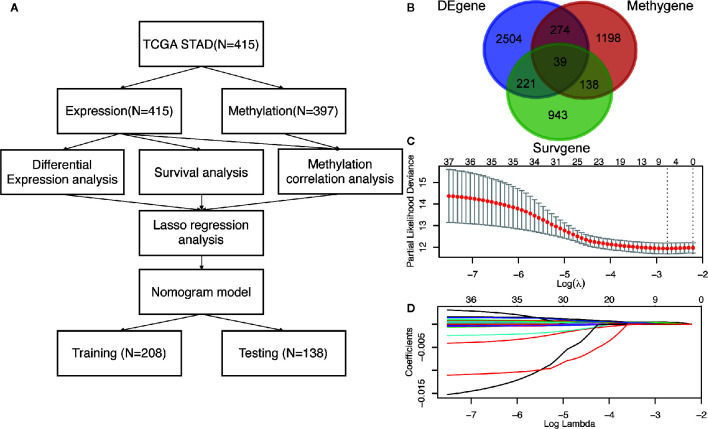
The identification of *DNA methylation-driven genes in GC*. **(A)** The work-flow in this study. **(B)** The Venn diagram of the DEgenes, Surgenes and Methygenes in 415 GC patients, of which 39 DNA methylation-driven genes are identified to be associated with overall survival of patients with GC. DEgenes, differentially expressed genes; Surgenes, survival-associated genes; Methygenes, methylation-associated genes. **(C)** 1000 iterations of Cox LASSO regression with 10-fold cross-validation is conducted to reduce the number of DNA methylation-driven genes. **(D)** A total of 5 DNA methylation-driven genes with nonzero coefficients are selected as candidate predictors.

The workflow of this study is presented in **Figure 1A**. In the TCGA dataset, a total of 415 GC patients enrolled with a median age of 67 years (range: 30 to 90 years). In general, 268 patients (65%) were male and 147 (35%) were female. There were 57 stage I (14%), 123 stage II (30%), 171 stage III (41%), 41 stage IV (10%) cases and 23 unknowns (5%). As to the Lauren classification, 176 patients (42%) and 72 patients (17%) were diagnosed with intestinal GC and diffuse GC, respectively. 373 patients had overall survival time more than 30 days with a median of 467 days (range: 31 to 3720 days). The detailed clinicopathological characteristics of 415 patients were shown in **Table 1** and **Table S1**.

**Table 1:** The clinicopathological characteristics of patients (N = 415).

Characteristics	Number (%)
Age	
>60	285 (69)
<= 60	130 (31)
Gender	
Female	147 (35)
Male	268 (65)
Stage	
I	57 (14)
II	123 (30)
III	171 (41)
IV	41 (10)
NA	23 (6)
Lauren classification	
Diffuse	69 (17)
Intestinal	176 (42)
Others	170 (41)
Race	
White	260 (63)
Asian	87 (21)
Black	12 (3)
Unknown	56 (13)
Radiotherapy	
Yes	72 (17)
No	303 (73)
Unknown	40 (10)

### Screening of DNA Methylation-Driven Genes in GC

To screen the driver genes in GC to achieve high prediction efficiency of the prognostic model, differential expression analysis, survival analysis and methylation correlation analysis were performed in the GC patients from TCGA cohort. Firstly, differential expression analysis was performed in mRNA expression data in GC samples (n = 415) and normal samples (n = 35) from TCGA. DEgenes were defined as the adjusted p value (p-adjust) < 0.05 and |log_2_ fold change (FC) | > 1. As results, 3038 DEgenes, including 2351 upregulated genes and 687 downregulated genes, were identified and used in further analysis (**Figure 1B** and **Table S2**). Secondly, survival analysis by univariate COX regression were performed in 373 GC patients with OS longer than 30 days. 1341 Surgenes were filtered with the criteria of p < 0.001 (**Figure 1B** and **Table S3**). Thirdly, correlation analysis between the expression level and the methylation value was performed in 397 GC patients and 1649 Methygenes were identified with |Coef|>0.5 and p <0.001 in GC patients (**Figure 1B** and **Table S4**). To screen out the candidate genes, Venn diagram demonstrated that 39 genes were significant in the differential expression, survival and correlation analysis (**Figure 1B** and **Table S5**). To further narrow down the candidate genes, we performed LASSO regression to eliminate numbers of variables which contributed less to the model (**Figure 1C**). The ability of these genes to predict prognosis was represented by the absolute value of their nonzero coefficients. Finally, a total of 5 DNA methylation-driven genes with nonzero coefficients were selected as candidate predictors (**Figure 1D**).

**Figure 2:**
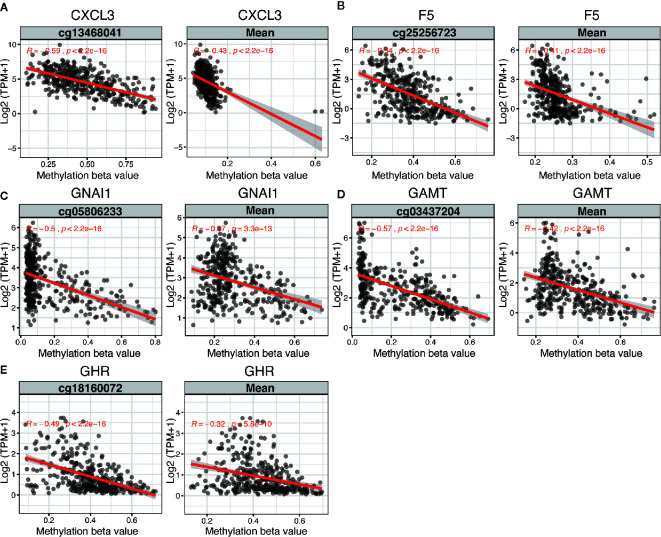
Regression analysis between gene expression and DNA methylation of five candidate predictors in TCGA dataset. Regression analysis between gene expression and DNA methylation (including methylation of CpGs in the promoter and mean methylation) of CXCL3 **(A)**, F5 **(B)**, GNAI1 **(C)**, GAMT **(D)** and GHR **(E)**. The horizontal and vertical axis represents methylation and mRNA expression of the DNA methylation-driven gene, respectively. TPM, transcripts per million.

The 5 DNA methylation-driven genes were C-X-C motif chemokine ligand 3 (*CXCL3*), coagulation factor V (*F5*), G protein subunit alpha I1 (*GNAI1*), Guanidinoacetate N-Methyltransferase (*GAMT*) and growth hormone receptor (*GHR*) and were selected to build the prognostic model. Correlation analyses of mRNA expression level and methylation level at representative CpG site and mean level of all CpG sites (CpGs) of the 5 genes were presented in **Figure 2**, the expression of *CXCL3*, *F5*, *GNAI1*, *GAMT* and *GHR* were all negatively correlated with the DNA methylation levels and were all statistically significant (**Figure 2** and **Table S6**).

### Prognostic Value of DNA Methylation-Driven Genes

**Figure 3:**
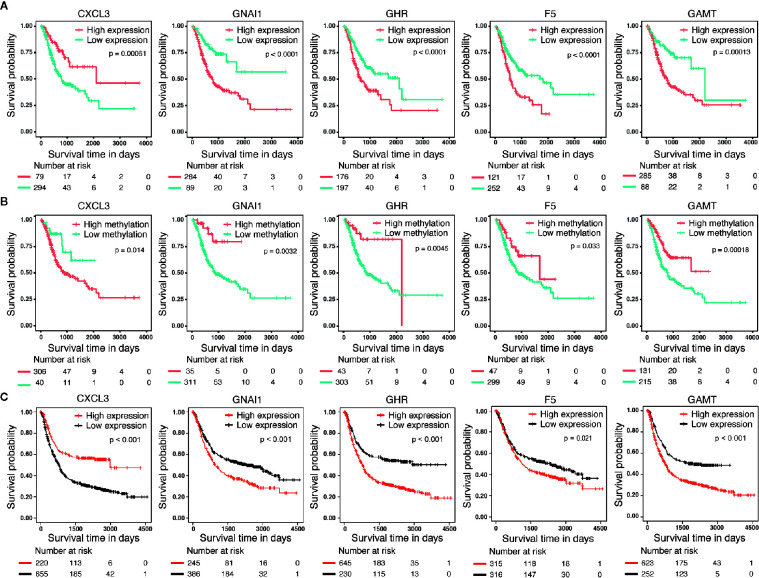
Survival analysis for five candidate predictors. **(A)** Survival analysis based on gene expression level for CXCL3, F5, GNAI1, GAMT and GHR in 373 patients with GC. **(B)** Survival analysis based on DNA methylation level for CXCL3, F5, GNAI1, GAMT and GHR in 346 patients with GC. The horizontal axis and vertical axis denote the survival time and the survival probability, respectively. **(C)** Survival analysis based on gene expression level for CXCL3, F5, GNAI1, GAMT and GHR in GSE14210, GSE15459, GSE22377, GSE29272, GSE51105 and GSE62254 microarray dataset.

We performed survival analyses based on different mRNA expression and DNA methylation levels of *CXCL3*, *F5*, *GNAI1*, *GAMT* and *GHR* in the dataset from TCGA. High expression and low DNA hypermethylation of *F5*, *GNAI1*, *GAMT* and *GHR*, as well as low expression and high DNA hypomethylation of *CXCL3* were significantly associated with worse prognosis (**Figures 3A, B** **)**, which demonstrating a negative regulatory relationship between DNA methylation and gene expression, suggesting the DNA methylation might be driven factor in these aberrantly expressed genes in GC. In addition, we observed the similar difference of outcomes between high expression and low expression patients in 6 microarray datasets, which validated the prognostic value of these DNA methylation-driven genes in GC (**Figure 3C**).

### Generation of the Risk Score and Prognostic Risk Model of Nomogram

**Figure 4:**
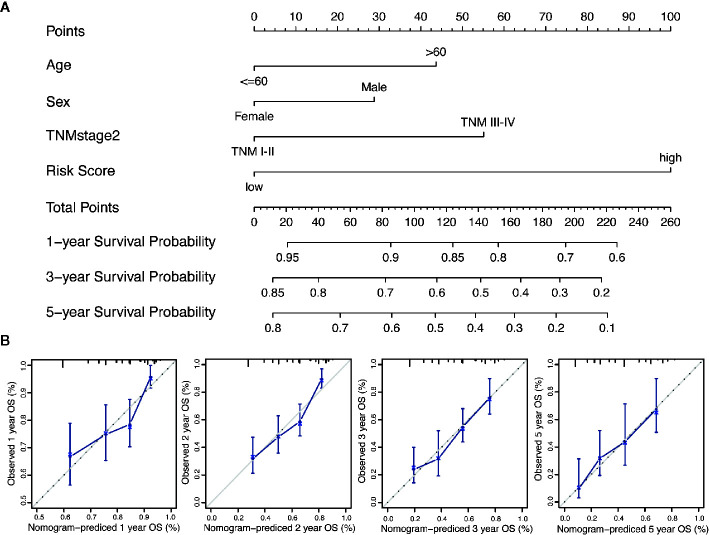
Generation of the nomogram composed of prognostic risk model and clinicopathological characteristics. **(A)** A quantitative nomogram is integrated using the risk score and available clinical characteristics for predicting the probability of 1-, 3-, and 5-year survival times for patients with GC. **(B)** The calibration plot of the nomogram between the predicted probabilities of survival and the 45-degree line at 1, 2, 3, and 5 years. The 45-degree line represents the best prediction.

We performed the multi-Cox proportional hazards regression analysis to calculate the risk score by the predictive function within R. Here, the cutoff value for the risk score was 0, which risk score < 0 represented low risk whereas risk score ≥ 0 represented high risk. GC patients were divided into high-risk and low-risk group according to the optimum cutoff. Next, we integrated the above prognostic risk model and available clinicopathological characteristics (age, gender and TNM stage) to build a quantitative nomogram for predicting the individualized probability of survival times in clinical practice (**Figure 4A**). The C-index for the nomogram was 0.674 (95% CI: 0.637–0.711), which showed a high predictive accuracy. The calibration curves of the nomogram between the predicted 1-, 3-, and 5-year OS probabilities and the best prediction (represented by the 45-degree line) showed good consistency (**Figure 4B**).

**Figure 5:**
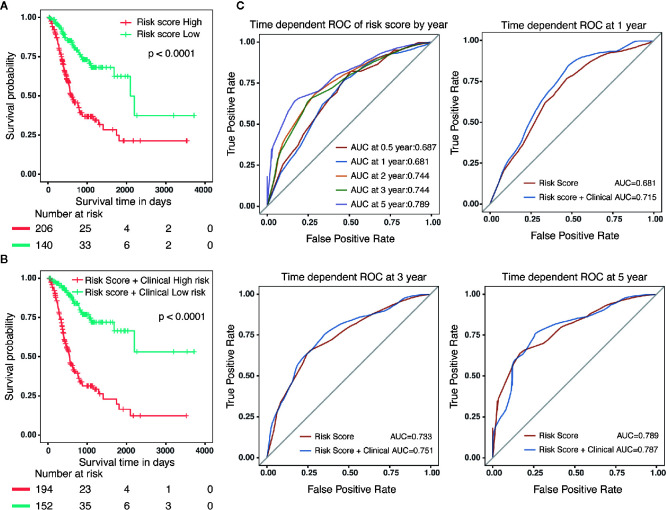
Validation of the prognostic risk model and nomogram. **(A)** Survival analysis in GC patients with high-risk and low-risk. HR = 0.325, 95% CI = 0.218–0.484, P < 0.001. **(B)** Survival analysis in GC patients with different level in risk score combined with clinical features. HR = 0.212, 95% CI = 0.139–0.322, P < 0.001. **(C)** The time-dependent area under the curves (AUCs) for 0.5, 1, 2, 3, and 5-year OS rates for GC patients, respectively.

Specifically, compared with patients with high-risk, low-risk patient showed significantly better OS (HR = 0.325, 95% CI = 0.218–0.484, p < 0.001) (**Figure 5A**). Combined with clinicopathological parameters, further validation of the prognostic efficacy for the nomogram showed that patients with high scores in both risk model and clinical features had a remarkedly worse OS (HR = 0.212, 95% CI = 0.139–0.322, p < 0.001) than that of low scores group (**Figure 5B**). In addition, ROC analysis showed that the time-dependent area under the curves (AUCs) for 0.5, 1, 2, 3, and 5-year OS rates for GC patients were 0.687, 0.681, 0.744, 0.744 and 0.789, respectively (**Figure 5C**). Specifically, the AUCs of the nomogram at 1 year, 3 year and 5 year were 0.715, 0.751 and 0.787, respectively. whereas the AUCs of risk score (0.681 at 1 year, 0.733 at 3 year and 0.789 at 5 year) were no less than that of risk score in combination with clinical features. Although the prognostic risk score itself had compatible predict efficiency similar to the nomogram in GC, the nomogram had a better clinical application prospect (**Figure 5C**).

### Validation of the Prognostic Nomogram

**Figure 6:**
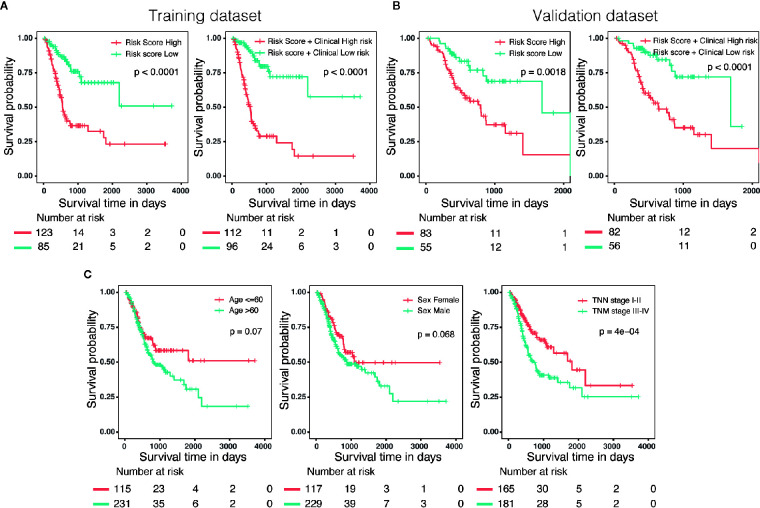
External validation of the risk model and nomogram. **(A)** Survival analysis of patients with different risk level in training dataset (N = 208). **(B)** Survival analysis of patients with different risk level in validation dataset (N = 138). **(C)** The predictive ability of the associated clinical features (age, gender and TNM stage) in 346 patients.

Three hundred forty-six GC patients with matching clinical information and OS more than 30 days were randomly stratified into a training dataset (N = 208) and a validation dataset (N = 138) by 6:4 ratio. Patients were classified into low-risk and high-risk groups utilizing the same cutoff value as previous analysis. Survival analysis revealed that patients with high scores in risk model or in nomogram had a significantly shorter OS than these in low score group (**Figures 6A, B** **)**. In addition, we also evaluated predictive ability of the associated clinical features (age, gender and TNM stage) in 346 patients, of which only the TNM stage was a significant prognostic predictor (**Figure 6C**), suggesting that the prognostic model of nomogram was much more powerful than clinicopathological parameters.

## Discussion

GC is the leading cause for cancer disability-adjusted life-years and accounts for 10% of the total worldwide (19). In recent years, despite important advances in early diagnosis and treatment options, and a slight decline in incidence and mortality, the burden of GC remains high (2). GC is a multistep disease and characterized by high heterogeneity, which involves numerous genetic and epigenetic variations. Previous research revealed that aberrant DNA methylation of tumor-associated genes might contribute to early detection of carcinogenesis and prediction of clinical outcome (10, 13, 20). Hence, the exploration and validation of DNA methylation-driven genes is necessary for early diagnosis and prognosis. In the present study, we integrated the paired transcriptomic and DNA methylation profiles to screen genes driven by methylation, and construct a prognostic nomogram for GC patients.

Hu CG et al. reported a risk assessment model based on expression of three *CLIP4* DNA methylation-associated genes in 393 GC samples from TCGA database (14). *CLIP4* was reported to regulate the expression of several genes associated with tumor invasiveness and metastasis, and the promoter methylation of *CLIP4* might be involved in the pathogenesis of GC. They identified 35 differently expressed genes between *CLIP4* hyper-methylation and hypo-methylation groups, of which *CLDN11*, *APOD* and *CHRDL1* were significantly associated with survival in GC patients (14). They further established a risk assessment model based on expression of *CLDN11*, *APOD* and *CHRDL1* for GC patients. The Univariate Cox regression analysis showed that targeted molecular therapy (HR = 0.6886, p = 0.0300), radiotherapy (HR = 0.4544, p = 0.0013) and risk value (HR = 0.4635, p = 0.0089) were significantly associated with overall survival time. However, whether other methylation-associated genes are involved in the initiation and progression of GC remains to be further explored. Recently, Long JY et al. integrated methylation and paired gene expression profiles of DEGs to identify DNA methylation-driven genes in 371 samples *via* MethylMix and LASSO analysis, and further built a risk score predictive model for patients with hepatocellular carcinoma based on expression of two DNA methylation-driven genes, which provided us novel insight (12).

In this study, we identified 3,038 DEgenes, 1,649 Methygenes, and 1,341 Survgenes *via* high throughput profiles and clinicopathological information of 415 GC patients from TCGA. Among these genes, a total of 39 genes were selected as candidate genes. Subsequently, we conducted univariate Cox regression analyses and LASSO regression of these 39 candidate genes. As a penalized regression method that uses an L1 penalty to shrink regression coefficients toward zero, LASSO analysis contributes to eliminate the number of variables and enhance the prediction accuracy (17). During 1000 iterations of Cox LASSO regression, the higher the nonzero coefficients of a gene presents, the stronger is the ability to predict prognosis. As a result, we identified five predictors (*CXCL3*, *F5*, *GNAI1*, *GAMT* and *GHR*), whose aberrant expression might be driven by DNA methylation. The prognostic efficacy showed that patients with high scores in risk score (HR = 0.325, 95% CI = 0.218–0.484, p < 0.001) and in nomogram ((HR = 0.212, 95% CI = 0.139–0.322, p < 0.001)) had worse OS than that of low scores group. Compared with the risk model based on expression of *CLIP4* DNA methylation-associated genes, our nomogram has a better performance in prognostic efficacy.

We observed a negative regulatory relationship between DNA methylation and mRNA expression of these predictors. Among them, low expression and high DNA hypomethylation of *CXCL3* were significantly associated with poor prognosis. CXCL3 is a family member of CXC chemokine ligand (CXCL). Previous research revealed that CXCL7 and CXCL1 were associated with the malignant progression of GC *via* CXCR2 signaling (21). Besides, a recent study identified *CXCL3* and *CXCL8* as diagnostic and prognostic genes in colon adenocarcinoma *via* integrating mRNA expression and DNA methylation profiles (22). The biological functions of CXCL3 in the initiation and progression of GC remains to be further validated.

In this study, *F5*, *GNAI1*, *GAMT* and *GHR* were poor prognostic factors in this study. F5 was a procofactor in the blood coagulation cascade, it functioned as a cofactor which activated coagulation factor X to convert prothrombin to thrombin (23). Previous research revealed that the high expression of *F5* was associated with improved overall survival in triple-negative breast cancer (24). Recently, the high expression of *F5* was reported to be significantly associated with a shorter OS in GC patients, which was consistent with our results (25). GHR was a member of the class I cytokine receptor family which played key roles in cancer progression. GHR was recently reported to mediate cell progression and apoptosis *via* the BRAF/MEK/ERK signaling pathway in breast cancer (26). According to the literature, GHR was elevated in GC serum samples and high expression of *GHR* mRNA was associated with a poor outcome in GC patients (27, 28), which suggested that GHR may serve as novel biomarkers for the early diagnosis and prognosis determination of GC.

GNAI1 belonged to the Gαi family, which primarily functioned as inhibitors of adenylyl cyclase. GNAI1 was reported to confer hydrogen peroxide-induced apoptosis in human lung cancer cells and be associated with the prognosis of thyroid cancer patients (29, 30). However, the biological functions of GNAI1 in GC remained unclear. GAMT was a new p53 target which connects p53 to creatine metabolism in the regulation of ATP homeostasis. GAMT was involved in p53-mediated genotoxic and metabolic stress-induced apoptosis (31). Previous research reported that *GAMT* expression was associated with the prognosis of GC patients received chemotherapy (32). In the present study, high expression and low DNA hypermethylation of *GAMT* was significantly associated with poor prognosis rates.

Nomograms have been widely used for cancer prognosis, resulted from their ability to transfer statistical predictive models into numerical estimate of the probability of death or recurrence. In this study, we constructed a prognostic nomogram based on expression of five DNA methylation-driven genes and clinicopathological parameters. Survival analysis showed that patients with high scores in nomogram had a significantly shorter OS than these in low score group, both in the training cohort and the validation cohort. Although the risk score itself had compatible predict efficiency similar to the nomogram in GC, the nomogram had a better clinical application prospect.

However, several limitations exist in this study. First of all, we focus on the integration of bioinformatics dataset to construct a prognostic risk model, which remains experimental validation in the future. Secondly, due to the limited size of samples with paired gene expression and DNA methylation data, we failed to validate the relationship between mRNA and methylation level in other databases of GC. At last, the nomogram was generated without clinical characteristics such as differentiation degree, Lauren classification, status of microsatellite instability (MSI) and tumor mutation burden (TMB), due to the incomplete information of GC patient in TCGA dataset.

## Data Availability Statement

All data relevant to the study are included in the article or uploaded as **Supplementary Material**.

## Author Contributions

ZC and XY conceived and designed the study. ZC and XY wrote and revised the manuscript. BL, MY, and HQ were responsible for the supplement and data analysis. All authors contributed to the article and approved the submitted version.

## Conflict of Interest

The authors declare that the research was conducted in the absence of any commercial or financial relationships that could be construed as a potential conflict of interest.
